# Spatio-temporal analysis of infant mortality in the city of Rio de Janeiro, 2010–2019

**DOI:** 10.1590/1984-0462/2022/40/2021061IN

**Published:** 2022-06-10

**Authors:** Viviane Gomes Parreira Dutra, João Roberto Cavalcante Sampaio, Camila de Souza Caputo, Raphael Mendonça Guimarães

**Affiliations:** a Universidade Estácio de Sá, Rio de Janeiro, RJ, Brazil.; b Universidade do Estado do Rio de Janeiro, Rio de Janeiro, RJ, Brazil.; c Escola Nacional de Saúde Pública Sérgio Arouca, Rio de Janeiro, RJ, Brazil.

**Keywords:** Infant mortality, Mortality excess, Social vulnerability, Spatio-temporal analysis, Mortalidade infantil, Excesso de mortalidade, Vulnerabilidade social, Análise espaçotemporal

## Abstract

**Objective:**

To describe the spatio-temporal distribution of infant mortality and its components in the city of Rio de Janeiro, Brazil, in 2010 and 2019.

**Methods:**

Infant mortality rate and the neonatal and postneonatal components were estimated. The standardized mortality rate was calculated to detect excess child mortality in the planning areas. Poisson regression was performed to estimate the effect of these planning areas on the years 2010 and 2019. Spatial analysis per neighborhoods was performed to identify the spatial autocorrelation rates, using the Moran’s Index and local indicator of spatial association (LISA).

**Results:**

The planning areas are very heterogeneous, depending on the history and evolution of occupation. There is an excess of mortality in planning areas with greater social vulnerability. In the Poisson model, it was observed that in all components, the planning area (PA) of residence was statistically significant as well as the year. Moran’s index did not show global spatial autocorrelation. However, when applying the LISA method, autocorrelation was observed at the local level and spatial clusters in the municipality of Rio de Janeiro.

**Conclusions:**

The spatial heterogeneity of the infant mortality rate in Rio de Janeiro suggests that local health policy strategies of each region consist in an efficient measure for reducing this rate.

## RESUMO

**Objetivo:**

Descrever a distribuição espaçotemporal da mortalidade infantil eseus componentes no município do Rio de Janeiro nos anos de 2010 e 2019.

**Métodos:**

Estimamos a taxa de mortalidade infantil e os componentes neonatal e pós-neonatal. Calculamos a taxa de mortalidade padronizada para detectar excesso de mortalidade infantil nas áreas de planejamento e realizamos regressão de Poisson para estimar o efeito dessas áreas nos anos de 2010 e 2019. Efetuamos análise espacial por bairros para detectar autocorrelação espacial das taxas, com uso do índice de Moran e do indicador local de associação espacial (LISA).

**Resultados:**

As áreas de planejamento são muito heterogêneas em função da história e da evolução da ocupação. Há excesso de mortalidade nas áreas de planejamento com maior vulnerabilidade social. No modelo de Poisson, observamos que em todos os componentes a área de planejamento de residência teve significância estatística, assim como o ano. O índice de Moran não mostrou autocorrelação espacial global. Contudo, ao aplicarmos o método LISA, observou-se autocorrelação em nível local e aglomerados espaciais no município do Rio de Janeiro.

**Conclusões:**

A heterogeneidade espacial da taxa de mortalidade infantil no Rio de Janeiro sugere que estratégias locais de políticas de saúde para cada região são uma medida eficiente para sua redução.

## INTRODUCTION

In many countries worldwide, infant mortality has been showing a downward trend, but this reduction is heterogeneous, with greater reductions in European countries and smaller reductions among countries in Sub-Saharan Africa and Oceania.^1^ In Brazil, the period from 1990 to 2015 was marked by progressive reductions in the infant mortality rate (IMR), which went from 47.1 to 13.4 per thousand live births, with the country reaching half the goal established by the United Nations as one of the Eight Millennium Development Goals ahead of the 2015 deadline.^2^

The IMR is considered an important indicator of the health status of a population, which reflects the general conditions of life, social well-being and economic development, access to and quality of care available in healthcare services of maternal and child health, especially primary health care.^3^ Thus, infant mortality is deemed a key indicator of human well-being and development, and the behavior of the rate at local scales can describe health inequalities and territorial disparities, becoming a monitoring indicator for the health of the entire population.^4^

In the last decade, Brazilian authorities have developed a series of strategies to improve access to health services and health indicators for the entire population. The expansion of primary health care through the Family Health Strategy (FSH) program is particularly noteworthy.^5^ Specially as of the end of 2009, the municipality of Rio de Janeiro, Brazil, induced the expansion of the FHS to carry out the Reform of Primary Health Care in the city.^6^ As a result, there was an increase in family health coverage from 6.9% in December 2009 to 70% at the end of 2016.7 This expansion allows for the reduction of the determinants of infant mortality, such as the improvement in the quality of health care and access to prenatal care and child health, contributing to the reduction of social, biological, and structural vulnerabilities.8

In recent years, there has been a significant decrease in IMR, but this reduction has not equally occurred in its components. The postneonatal component shows a tendency to decline, whereas the neonatal component corresponds to more than 70% of deaths in the first year of life.^9^ However, it is known that most deaths in the neonatal period are preventable, such as those resulting from prematurity and low birth weight, which demonstrates the importance of improving the quality of care in pregnancy, childbirth and the puerperium.^10^

The municipality of Rio de Janeiro has an estimated population of 6,747,815 inhabitants for 2020^11^ and is administratively divided into ten planning areas (PAs). Each PA has distinctive characteristics that define the priorities of health actions as well as the coverage of the FHS. Area 1.0 is the historic center of the city, with predominantly urban space, and receives people from other municipalities that use the services of the health network established in the Center. Area 2.1 includes neighborhoods with higher standards of living, better per capita income, longevity, and education level. Even the slum regions in this area have a different profile from the more peripheral areas of the municipality. Area 2.2 has a population of residents with high literacy rate between eight and nine years of study, higher than the average for the city. This area is surrounded by several communities, housing a universe of 69,031 inhabitants, and only Andaraí neighborhood is not covered by the FHS. Area 3.1 has neighborhoods with different levels of development, which occupy the extremes of the rankings of the human development index (HDI) and the social development index (SDI) of the municipality. Area 3.2 presents irregular occupation, with urban areas interspersed with subnormal agglomerates, and is composed of several communities. Area 3.3 is characterized by having large commercial centers, industrial centers, and numerous areas of irregular occupation and disorderly advance. Area 4.0 has an important demographic growth rate and the literacy rate of residents is higher than the average for the city. It has neighborhoods with a population of high average income per capita. Area 5.1, located approximately 40 km from the central region of Rio de Janeiro, is geographically the beginning of the western zone of the municipality, a place with a notable decrease in socioeconomic indicators. Area 5.2 is the most populous in the city and has a low HDI. Finally, area 5.3 is located in the westernmost region of the municipality of Rio de Janeiro, far from the central region of the city. Approximately 45% of the population lives in irregular, clandestine, and slum-like areas.

Although the municipality of Rio de Janeiro has expanded its coverage of primary health care, the city is endowed with great urban heterogeneity. This territorial disparity is reflected not only in the coverage itself, but in a set of social indicators, in such a way that inequality in health remains an important challenge for health managers.^12^ With the expansion of the FHS in the city, the assessment and monitoring of health indicators are necessary, which represents a possibility for the management and development of preventive strategies, including those aimed at reducing the risk of death in children under one year of age. Taking this into consideration, the objective of this study was to describe the spatio-temporal distribution of infant mortality and its components in the city of Rio de Janeiro, Brazil, in 2010 and 2019.

## METHOD

This is an ecological study whose units of analysis were the neighborhoods of the municipality of Rio de Janeiro. The municipality is divided into 160 neighborhoods and ten PAs and had an estimated population of 6,747,815 inhabitants in July 2020.^12^ These areas are quite heterogeneous in terms of socioeconomic characteristics and properly characterize the considerable intra-urban inequality of the city. In this sense, to characterize these areas, data from the initial year of observation (2010) were used, considering social and demographic indicators and their respective qualifications.

Deaths of children under one year of age in the period from 2010 to 2019 were taken into account, and they were selected based on information from the Death Certificate.

The IMR was calculated according to the formula:


$$\text{IMR}=\frac{\text{number of deaths among children under 1 year of age}}{\text{number of live births}}\times 1,000$$

Hence, the IMR was divided into neonatal infant mortality (up to 27 days of birth) and postneonatal mortality (28 to 364 days of birth).

Based on the characterization, the mortality rates in PAs in the initial and final years of the series were described. Subsequently, the rate variation was calculated per PAs, between 2010 and 2019. Moreover, with the purpose of comparing PAs, the standardized mortality ratios were estimated to identify excess infant mortality per PA, assuming that, to define the expected number, the IMR would be the same for the municipality of Rio de Janeiro altogether.^13^ To do so, the value of the standardized mortality ratio (SMR) and the 95% confidence interval (95%CI) were calculated according to the following formula:


$${\text{Lower limit: e}}^{\left[\ln(\text{SMR})-{\text{Z}}_{1-\text{α}/2\frac{1}{\sqrt{\text{obs}}}}\right]}$$

$${\text{Upper limit: e}}^{\left[\ln(\text{SMR})+{\text{Z}}_{1-\alpha /2}\frac{1}{\sqrt{\text{obs}}}\right]}$$

Finally, to assess whether there is an association between the observation units (neighborhoods and PAs), considering them a proxy of urban inequality in the city, the rates were compared in two stages, as described next.

The infant mortality count was estimated based on the occurrence of infant, neonatal, and postneonatal deaths for each PA in the municipality of Rio de Janeiro. To compare the ratio in each category of covariates, Pearson’s chi-square test and its respective p-value were used.^13^ To investigated the difference in IMR and fractions, the data were adjusted using a Poisson model. Crude and adjusted prevalence ratios were calculated according to the best model obtained by maximum likelihood estimation. Confidence intervals at the 95% significance level were also estimated.^14^

In order to assess the neighborhood as a unit of analysis, the global Moran’s index (Moran’s I) was calculated to assess the spatial autocorrelation of infant mortality rates and fractions, and the local indicator of spatial association (LISA) was estimated to locally identify clusters in the municipality that presented statistical significance.^15^

All analyses were carried out for the years 2010 and 2019, respectively, in such a way to enable the comparison of the magnitude of associations and spatial correlations between years. To perform the regression with the Poisson model, the R 4.0.0 software was used and, for the spatial analysis, the GeoDa software version 1.14.

## RESULTS

The PAs are very heterogeneous depending on the history and evolution of the occupation. PA 1 concentrates the highest proportion of people living in slums (29.0%). Conversely, the largest number of health service units installed in the city is concentrated in this area. PA 2.1 has the largest population of older adults (23.1%), the highest population density in the city (14,051 inhab./km^2^), the smallest proportion of children aged zero to 14 years (12.8%), and the highest municipal HDI. PA 2.2 is characterized by a profile very similar to that found in PA 2.1. The participation of the group of older adults in PA 2.2 is also considerable, the second largest in the city (22.1%). PA 3.1, 3.2 and 3.3 together are characterized as the most populous area of the city (37.9%), and half of the slum dwellers live in this region. PA 4 is the second largest in area, with 294 km^2^, or 1/4 of the territory of the state capital. This region is a vector of urban expansion for middle and high incomes. Nowadays, it has the second largest population (910 thousand inhabitants) and the lowest population density in the city (3,097 inhab./km^2^). Finally, PA 5.1, 5.2 and 5.3, in demographic terms, compose the second most populous area in the municipality, accounting for 27% of the city’s population. In other words, one out of every four residents of Rio de Janeiro lives in the west zone, which is a vector of urban expansion for middle- and low-income populations (Chart 1).

**Chart 1:** Social and demographic indicators of the municipality of Rio de Janeiro, 2010.

Indicator	Planning area									
	1.0	2.1	2.2	3.1	3.2	3.3	4.0	5.1	5.2	5.3
Dependency ratio	50	56	54.7	51.6	53	53.6	48	46,1	51	56
Aging index	76	172	152.8	85.7	106	75.9	65	54.9	55	42
Income ratio	2.7	2.5	2.5	1.8	2.1	2.1	2.6	2.3	1.8	2.0
Per capita average family income	522.7	1,806.1	1,396.8	484.8	672.9	500.1	684.2	384.1	389.5	300.3
SDI	0.6	0.7	0.7	0.6	0.6	0.6	0.6	0.6	0.6	0.5

SDI: Social Development Index.

Source: Instituto Pereira Passos, RJ, 2021.^12^

The SMR per component of infant mortality for each PA in the municipality of Rio de Janeiro in 2010 and 2019 is shown in Table 1. Infant mortality in PAs 3.3 and 5.3, areas where there is greater social vulnerability, showed excess deaths in 2010 of 21 and 19%, respectively, when compared with the municipality of Rio de Janeiro (SMR=1.21; 95%CI 1.07–1.37; and SMR=1.19; 95%CI 1.02–1.38). Conversely, in PAs 2.1 (SMR=0.64; 95%CI 0.51–0.80) and 2.2 (SMR=0.64; 95%CI 0.47–0.87), which have a profile with better performance of social indicators, infant mortality was lower. For the year 2019, it can be observed that PA 3.3 presents an increase in excess deaths of 33% compared with 2010 (SMR=1.33; 95%CI 1.16–1.51). Excess deaths were also observed for PA 5.3 (SMR=1.28; 95%CI 1.07–1.53). Such were not observed in 2010. In the neonatal period, there were excess deaths in PAs 3.3 and 5.1 (SMR_PA3.3_=1.21; 95%CI 1.04–1.41; SMR_PA5.1_=1.33; 95%CI 1.11–1.58) in 2010. Conversely, when analyzing the year 2019, PAs 3.1 and 5.3 show excess deaths (SMR_PA3.1_=1.23; 95%CI 1.02–1.48; SMR_PA5.3_=1.37; 95%CI 1.09–1.71). On the one hand, in the postneonatal component, in 2010, all PAs had mortality similar to that expected. On the other hand, in 2019, there was an increase in excess deaths of 44% in PA 3.3 compared with 2010 (SMR=1.44; 95%CI 1.16–1.79).

**Table 1:** Standardized mortality ratios per component of infant mortality, according to planning area. Municipality of Rio de Janeiro, 2010 and 2019.

Component	PA	2010			2019		
		SMR^†^	95%CI		SMR^†^	95%CI	
			LL	UL		LL	UL
Neonatal	1.0	1.09	0.84	1.41	1.08	0.79	1.47
	2.1	0.59	0.44	0.79	0.64	0.45	0.90
	2.2	0.66	0.45	0.97	0.92	0.62	1.36
	3.1	0.93	0.77	1.12	1.23	1.02	1.48
	3.2	0.82	0.64	1.04	0.99	0.76	1.28
	3.3	1.21	1.04	1.41	1.41	1.19	1.66
	4.0	0.99	0.85	1.17	0.84	0.69	1.01
	5.1	1.03	0.81	1.31	1.03	0.82	1.30
	5.2	1.01	0.84	1.22	1.03	0.85	1.26
	5.3	1.33	1.11	1.58	1.37	1.09	1.71
Postneonatal	1.0	0.87	0.59	1.28	1.35	0.94	1.96
	2.1	0.72	0.51	1.03	0.88	0.59	1.31
	2.2	0.60	0.35	1.02	0.69	0.37	1.27
	3.1	0.95	0.74	1.21	1.09	0.84	1.42
	3.2	1.09	0.82	1.44	0.97	0.68	1.38
	3.3	1.21	0.99	1.48	1.44	1.16	1.79
	4.0	1.04	0.84	1.29	0.73	0.56	0.96
	5.1	0.93	0.71	1.23	1.15	0.86	1.54
	5.2	1.00	0.77	1.29	1.02	0.78	1.33
	5.3	1.23	0.92	1.66	1.37	1.01	1.85
Infant	1.0	1.01	0.81	1.25	1.10	0.87	1.39
	2.1	0.64	0.51	0.80	0.68	0.52	0.88
	2.2	0.64	0.47	0.87	0.78	0.56	1.09
	3.1	0.94	0.81	1.09	1.10	0.95	1.29
	3.2	0.92	0.76	1.10	0.92	0.74	1.13
	3.3	1.21	1.07	1.37	1.33	1.16	1.51
	4.0	1.01	0.89	1.15	0.75	0.64	0.87
	5.1	1.19	1.02	1.38	1.00	0.84	1.20
	5.2	1.01	0.87	1.17	0.96	0.82	1.13
	5.3	1.10	0.92	1.33	1.28	1.07	1.53

PA: planning area; 95%CI: 95% confidence interval; SMR†: standardized mortality ratio, LL: lower limit; UL: upper limit.

When investigating the association between the components of the IMR, PAs and the year, it was observed that both the PA of residence and the year had statistical significance in all components. In the adjusted model, the year 2019 showed a reduction in IMR and in all its components when compared with the year 2010. This decrease was similar for all components.

When analyzing the PAs, there was statistical significance in all components. However, it is worth highlighting that infant mortality in PAs 2.1, 2.2 and 4.0, which have better performance in the main social indicators, presented a rate ratio (RR) lower than 1 and statistically significant (respectively: PA 2.1 RR=0.65, 95%CI 0.54–0.78; PA 2.2 RR=0.70, 95%CI 0.55–0.88; PA 4.0 RR=0.88, 95%CI 0.79–0.98) when compared with the IMR of the city of Rio de Janeiro. Conversely, PAs 3.3 and 5.3, which present the worst performance in social indicators, show a rate ratio greater than 1 and statistically significant (respectively: PA 3.3 RR=1.27, 95%CI 1.15-1.39; PA 5.3 RR=1.18, 95%CI 1.03–1.35), which indicates that IMR was higher in this area than in the city. The neonatal and postneonatal components showed the same association for PAs 2.1 and 2.2. For PA 4.0, there was no statistical significance for the components separately. The pattern obtained for PAs 3.3 and 5.3 in infant mortality was repeated for the components, separately, only in PA 3.3 (Table 2).

**Table 2:** Crude and adjusted Poisson regression models for infant mortality and its components and covariates. Municipality of Rio de Janeiro, 2010 and 2019.

Component			Crude model				Adjusted model			
			RR	95%CI		p-value	RR	95%CI		p-value
				LL	UL			LL	UL	
Neonatal	PA	MRJ	1.00				1.00			
		1.0	1.06	0.86	1.29	<0.001	1.05	0.85	1.28	<0.001
		2.1	0.59	0.47	0.74		0.59	0.47	0.74	
		2.2	0.76	0.57	0.99		0.75	0.56	0.98	
		3.1	1.03	0.90	1.18		1.03	0.89	1.18	
		3.2	0.87	0.72	1.04		0.87	0.72	1.04	
		3.3	1.26	1.12	1.42		1.26	1.12	1.42	
		4.0	0.89	0.78	1.02		0.90	0.78	1.02	
		5.1	1.17	1.00	1.35		1.16	1.00	1.34	
		5.2	0.99	0.85	1.13		0.99	0.86	1.14	
		5.3	1.15	0.96	1.36		1.15	0.97	1.36	
	Year	2010	1.00				1.00			
		2019	0.88	0.83	0.95	<0.001	0.88	0.82	0.94	<0.001
Postneonatal	PA	MRJ	1				1			
		1.0	1.05	0.78	1.36	0.002	1.04	0.78	1.36	0.0002
		2.1	0.76	0.57	0.99		0.76	0.57	0.98	
		2.2	0.60	0.38	0.89		0.60	0.38	0.88	
		3.1	0.98	0.80	1.18		0.97	0.80	1.17	
		3.2	1.01	0.79	1.26		1.01	0.79	1.26	
		3.3	1.27	1.08	1.49		1.27	1.08	1.49	
		4.0	0.86	0.72	1.03		0.87	0.72	1.03	
		5.1	1.00	0.80	1.23		1.00	0.80	1.23	
		5.2	0.96	0.79	1.17		0.97	0.79	1.17	
		5.3	1.24	0.99	1.54		1.24	0.99	1.54	
	Year	2010	1				1			
		2019	0.87	0.80	0.96	0.005	0.87	0.80	0.96	
Infant mortality	PA	MRJ	1				1			
		1.0	1.05	0.89	1.23	<0.001	1.05	0.88	1.23	<0.001
		2.1	0.65	0.54	0.77		0.65	0.54	0.77	
		2.2	0.70	0.55	0.87		0.69	0.55	0.87	
		3.1	1.01	0.90	1.13		1.01	0.90	1.13	
		3.2	0.91	0.79	1.05		0.91	0.79	1.05	
		3.3	1.26	1.14	1.39		1.26	1.14	1.39	
		4.0	0.88	0.79	0.97		0.88	0.79	0.98	
		5.1	1.10	0.98	1.24		1.10	0.97	1.24	
		5.2	0.97	0.87	1.09		0.98	0.87	1.10	
		5.3	1.18	1.03	1.34		1.18	1.03	1.35	
	Year	2010	1				1			
		2019	0.88	0.83	0.93	<0.001	0.88	0.83	0.92	

PA: planning area; MRJ: Municipality of Rio de Janeiro; RR: rate ratio; 95%CI: 95% confidence interval; LL: lower limit; UL: upper limit.

**Figure 1:**
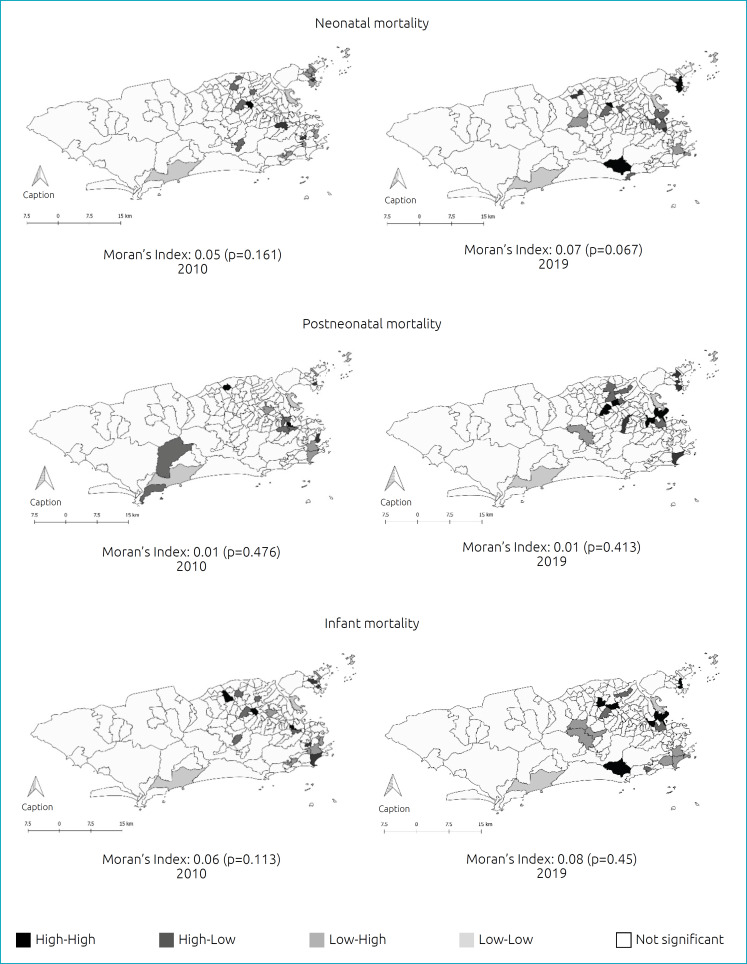
Global and local spatial autocorrelation of infant mortality and its components. Municipality of Rio de Janeiro, 2010 and 2019.

Control charts were prepared for residuals of the models. The modeling premise is that, when process data undergo autocorrelation, the assumption of residual independence is violated. For Poisson models, correlation graphs are frequently used for the analysis of the autocorrelation (ACF) and partial autocorrelation (PACF) functions. Through visual inspection, it is possible to observe if there are values that deviate from the expected band for the residual values. Whenever the values are within the bands, it means that the residuals are independent. No spatial correlation was identified in the residuals; therefore, the condition required to perform a spatial regression was not met. Thus, the present results are based on the analysis of spatial autocorrelation, an exploratory technique. Although Moran’s I does not show global spatial autocorrelation for the IMR and its components in the city of Rio de Janeiro (Figure 1), when employing the LISA method, autocorrelation was observed at the local level and spatial clusters in the city.

Neighborhoods that presented high-high IMR in 2010 (Cavalcanti, Guadalupe and Madureira) are located in PA 3.3. For the year 2019, this pattern was also observed in neighborhoods of PAs 3.1 (Cocotá, Itanhangá, Pitangueiras, Praia da Bandeira), 1.0 (Benfica, Caju), and 3.3 (Rocha Miranda, Vaz Lobo and Vicente de Carvalho). It was observed that neighborhoods with mortality rates classified as high-high are more prevalent in the northern zone of the city of Rio de Janeiro in both analyzed periods. The neighborhoods of Cascadura, Cavalcanti and Madureira had high-high early neonatal mortality rates in 2010. Conversely, for 2019, the neighborhoods that presented these rates were Piedade, Rocha Miranda and Vaz Lobo. For the late neonatal component, in 2010, the neighborhoods of Anchieta, Centro, and Ricardo de Albuquerque presented high-high spatial clusters. In 2019, this pattern was observed in the neighborhoods of Cacuia, Cocotá, Pitangueiras and Praia da Bandeira. For the postneonatal component, high-high spatial clusters were observed in 2010 in the neighborhoods of Costa Barros (PA 3.3) and Mangueira (PA 2.2); and in the neighborhoods of PAs 1.0 (Benfica and Caju) and 3.3 (Madureira, Rocha Miranda and Vicente de Carvalho) in 2019. Once again, a pattern in the spatial distribution of rates was observed, especially among neighborhoods with the worst performance in social indicators.

## DISCUSSION

Between 2010 and 2019, there was reduction in IMR and all its components in the city of Rio de Janeiro. This reduction in the municipality of Rio de Janeiro follows a trend observed in the country, as this is a pact and one of the goals of the Sustainable Development Goals (SDGs) established by the United Nations (UN) in 2017. It is noteworthy that in 2011 the country attained half of the established goal, 15.7 deaths per thousand live births, before the deadline set for 2015.^16,17^

Tracking the effect of a primary health intervention on access to health care is difficult due to the synergistic and overlapping effect of interventions and initiatives that aim to improve the well-being of the population.^4^ Nonetheless, primary health care has been recognized as a clinical strategy to improve the health status of the population in developing countries.^18^ This study presented descriptive statistics to illustrate the pattern of infant mortality in different intra-urban settings, characterized by social indicators that express the social vulnerability present in these places.

Although Rio de Janeiro has remarkably improved child health care and reduced infant mortality since 2010, geographic variations throughout the city and the small difference between the neonatal and postneonatal components highlight two different aspects of healthcare policies.

First, it is worth noting that economic factors and health resources are important factors that influence child mortality. Different circumstances and interventions carried out within the public sector contributed to the progress in child survival observed in Brazil in recent decades, including: (i) universalization of health care provided by the Brazilian Unified Health System (SUS), with a reduction in its inequalities in access and coverage; (ii) socioeconomic and demographic changes; (iii) conditional cash transfer programs; (iv) improvements in sanitation conditions; (v) breastfeeding promotion and immunization programs; and (vi) the implementation of many national and state programs to improve child health and nutrition.^17,19^ It is noteworthy that some of these predictors are not usually associated with direct medical care. Therefore, this inequality not only significantly improves with medical interventions, but also with broader and comprehensive socioeconomic interventions at national and regional levels.^20,21^

Second, it is worth considering that neonatal and postneonatal mortality have very different risk factors. The first component is strongly influenced by the quality of the provided health care, whereas the second is more directly related to socioeconomic and environmental determinants.^22^ From the 1990s onward, there has been a change in the predominance of components, and neonatal deaths have surpassed the postneonatal ones.^23^ Hence, it is worth highlighting the limitations of current public policies regarding the ability to detect fetal malformations, duration of gestation, and fetal growth during prenatal care, which is an important measure for reducing neonatal mortality.^24^ Although inhospitable socioeconomic conditions favor postneonatal mortality, they often indicate a context that also reflects in difficulty in accessing quality healthcare services, which is key to the explanation of neonatal mortality.^25^

Therefore, there are still challenges for the global reduction of infant mortality and the mitigation of important characteristics in the city of Rio de Janeiro, such as the concentration of deaths in the neonatal period and possible specific increases in postneonatal mortality, especially after the recent cuts in social investments.^3^ Some forceful interventions to reduce child mortality may be social and public health initiatives that mitigate disparities in sociodemographic and economic risks.^26^ The study conducted by Bonfim et al.,^27^ when analyzing infant deaths that occurred in Recife (state of Pernambuco, Brazil), found that social deprivation was associated with preventable infant deaths. Likewise, Ramalho et al.^28^ had already identified, years ago, a relationship between what they called the family development index, a socioeconomic indicator, and infant mortality in Brazilian municipalities.

The limitations of the present study are related to its design, not allowing inference of the results at the individual level, and the use of secondary data from health information systems, considering the quality of information in the available records. The use of data from the Brazilian Mortality Information System (*Sistema de Informações sobre Mortalidade* – SIM) may be related to underreporting of infant deaths, which results in misinformation about the actual number of deaths, leading to an underestimation of mortality rates. It is noteworthy that ecological studies are useful to identify both the levels of health of a population and to formulate research hypotheses for public health.

Finally, this study investigated the spatial heterogeneity at the municipal level and the clustering of infant mortality rates and their components in the city of Rio de Janeiro. Rates decreased between 2010 and 2019 for infant mortality and components. However, ultimately, socioeconomic and health inequalities are persistent. Despite the improvement in infant mortality indicators and their components, intra-urban differences are reflected in the difference in behavior of the indicators when compared with municipal indicators. The spatial heterogeneity of the distribution of socioeconomic factors and IMR in Rio de Janeiro suggests that local health policy strategies for each region may be an efficient measure to reduce infant mortality.
